# A new genus and species of deep-sea glass sponge (Porifera, Hexactinellida, Aulocalycidae) from the Indian Ocean

**DOI:** 10.3897/zookeys.136.1626

**Published:** 2011-10-13

**Authors:** Sabyasachi Sautya, Konstantin R. Tabachnick, Baban Ingole

**Affiliations:** 1 National Institute of Oceanography, Dona Paula, Goa, 403004, India; 2 Institute of Oceanology Ac. of Sc. of Russia, Nahimovsky 36, Moscow, 117997, Russia

**Keywords:** Porifera, Hexactinellida, Aulocalycidae, glass sponge, new genus, new species, Carlsberg Ridge, Indian Ocean

## Abstract

New hexactinellid sponges were collected from 2589 m depth on the Carlsberg Ridge in the Indian Ocean during deep-sea dredging. All fragments belong to a new genus and species, *Indiella* **gen. n.** *ridgenensis* **sp. n.**, a representative of the family Aulocalycidae described here. The peculiar features of this sponge, not described earlier for other Aulocalycidae, are: longitudinal strands present in several layers and epirhyses channelization.

## Introduction

**Figure 1.**
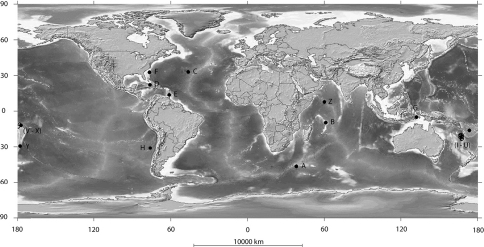
Global distribution of Aulocalycidae including the present study **A** *Aulocalyx irregularis* **B** *Aulocalyx serialis* **C–F** *Rhabdodictyon delicatum* **G** *Ijimadyctyum kurense* **H** *Leioplegma polyphyllon* **I–Y** *Euriplegma auriculare* **Z** *Indiella* gen.n. *ridgenensis* sp.n.

The family Aulocalycidae was established by Ijima (1927) for 5 genera (Fig. 1): *Aulocalyx* Schulze, 1886, *Rhabdodicyum* Schmidt, 1880, *Tretopleura* Ijima, 1927, *Euryplegma* Schulze, 1886 and *Fieldingia* Kent, 1870. One genus *Ijimadyctyum* Mehl, 1992 was raised from a previously known second species, *Rhabdodicyum kurense* Ijima, 1927. One genus was added later *Leioplegma* Reiswig & Tsurumi, 1996. Tabachnick and Reiswig (2000) ejected two genera: *Tretopleura* and *Fieldingia* form the family and a suggested a new order Aulocalycoida with a single reorganized family. A new subfamily Uncinateriinae with two genera: *Uncinatera*, Topsent, and *Tretopleura* were suggested by Reiswig (2002) as a subdivision of Aulocalycidae together with Aulocalycinae (with the scope and definition of former Aulocalycidae of Tabachnick and Reiswig (2000)). A new subfamily Cyathellinae of the family Aulocalycidae with the only genus *Cyathella* Schmidt, 1870 was suggested by Janussen and Reiswig (2003). The new genus, describing in this paper is a unquestionable representative of the family Aulocalycidae sensu Tabachnick and Reiswig (2000) and subfamily Aulocalycinae *sensu* Reiswig (2002).

## Taxonomy

### Family Aulocalycidae Ijima, 1927

#### Indiella gen. n.

urn:lsid:zoobank.org:act:DDD70A14-F35A-4F19-99A1-B58720834CF5

http://species-id.net/wiki/Indiella

##### Diagnosis.

 Fan (or funnel)-like basiphytous sponge with thin walls and numerous epirhyses. Framework contains several layers of regular dictyonal strands (mainly from the atrial side) and irregular fused hexactinic spicules (which form a typical aulocalycoid skeleton) located among them and from the dermal side. Dermalia and atrialia are pentactins. Microscleres are discohexasters.

##### Etymology.

The name of the genus is derived from its place of collection and refers to the Indian Ocean.

##### Definition.

 Aulocalycidae with fan (or funnel)-like body, epirhyses, and several regular layers of dictyonal strands located mainly on the atrial side.

##### Remarks.

 It is likely that the body is rather fan-like than cup or funnel-like since the fragments are flat, thus the funnel-like body shape should be of a very large diameter. The original shape of the body is already known in Aulocalycoidae: *Leioplegma* Reiswig & Tsurumi, 1996, while wide funnels are unknown. Basiphytous type of fixation to likely hard substratum is suspected since all other representatives of the family have it. The taxonomic affiliation of genus *Cyathella* (its attribution to the Aulocalycoida, Aulocalycidae with definition of a new subfamily Cyathellinae was made by Janussen and Reiswig 2003), possessing a rhizophytous type of fixation is unique for recent hexactinellids with rigid skeleton.

The walls in the new genus are relatively thick (in comparison with other representatives of the family). Usually the aulocalycoid skeleton is composed of large hexactins located approximately in a single layer, their rays are distributed in a single plane (the distal one and proximal are bent), fusion takes place at points of mutual contact, so the wall thickness includes an only dictyonal layer. The regular dictional strands are observed in *Leioplegma* only, they are present as a single layer of parallel units longitudinally distributed, and irregular aulocalycoid skeleton is situated among them (Reiswig and Tsurumi 1996). The walls in *Euryplegma* appear to be very complicated and their construction has no equivalent interpretation (Tabachnick and Reiswig 2000). *Cyathella* has similar framework construction with several layers of dictyonal strands, but it has no channels and likely no loose spicules.

The presence of epirhyses type of channelization is unique for the family. It is known in Euretidae (Hexactinosida), for instance, in *Chonelasma* (Reiswig & Wheeler, 2002). Among the other types of channelization in Aulocalycoidae, only schizorhyses-like ones are known in *Euryplegma*, meantime as in the case with complicated wall construction, they may be intercavaedia-like constructions between the atrial cavity and numerous small lateral oscula (Tabachnick and Reiswig 2000).

The loose spicules are typical for the family where few species possess scepters and uncinates. A more simplified spicule set is observed in *Heterochone* (Hexactinosida: Euretidae), which has no loose spicules other then discohexasters (Reiswig and Wheeler 2002).

The situation with aulocalycoid, paraulocalycoid and skeleton of *Cyathella*-like construction (Reiswig 2002; Janussen and Reiswig 2003) is becoming more complicated after finding in the dictional strands of *Farrea* numerous axial canals (Reiswig 2004), thus the definition of Aulocalycidae into subfamilies seems to be poorly established and the new genus is regarded as a representative of Aulocalycidae.

##### Type species.

 *Indiella ridgenensis* sp.n.

#### Indiella ridgenensis sp. n.

urn:lsid:zoobank.org:act:185CC226-9FF5-42C9-8EA1-999CB8EF1146

http://species-id.net/wiki/Indiella_ridgenensis

Figs 2 [Fig F3] 4 

##### Etymology.

 The species name is derived from its type locality, the ridge (Carlsberg Ridge) habitat.

##### Material examined.

 Carlsberg Ridge, Indian Ocean: ‘Akademic Bois Petrov’ station. DR-13, 07°00.466'N, 59°56.295'E, 2589 m, November 2009.

##### Holotype.

 NIO/BOD/5-H/2011, stored in ethanol. NIO/SPONGE/DR-13/H, slide, stored in ethanol. IORAS (Institute of Oceanology of Russian Academy of Sciences) 5/2/ NIO/BOD/5-H/2011 (slides).

**Paratypes**: NIO/BOD/5-P1, NIO/BOD/5-P2, NIO/BOD/5-P3, stored in ethanol. NIO/SPONGE/DR-13/Pi, NIO/SPONGE/DR-13/Pii, NIO/SPONGE/DR-13/Piii, slides. IORAS NIO/BOD/5-P1, NIO/BOD/5-P2, NIO/BOD/5-P3, slides.

##### Description.

**Figure 2.**
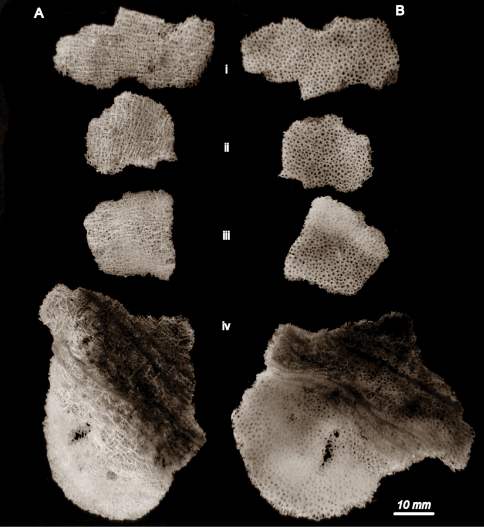
*Indiella* gen.n. *ridgenensis* sp.n. **A** view from the dermal side **B** view from the atrial side; (i) holotype, (ii) to (iv) paratypes

 **Body:** The sponge consists of small, lamellate, thin fragments. The holotype is a flat fragment approximately 40×17 mm about 1 mm in thickness (Fig. 2i). Paratypes are similar: Pi is a lamellum 20×25 mm (Fig. 2ii); Pii is 30×20 mm (Fig. 2iii); Piii is 50×45 mm (Fig. 2iv). From the dermal side numerous epirhyses are observed, they are 1.3 – 1.5 mm (Fig. 4C) in diameter and penetrate about a half of the wall thickness.

Spicules framework is seems to be constructed of different elements: regular, longitudinally directed dictyonal strands, located mostly in the vicinity of the atrial surface (approximately 4 layers) and irregular hexactins fused to each other and to the regular elements at points of mutual contacts, at all levels of the wall thickness. All framework surfaces are covered by very small spines, the free outer ray ends are conically pointed. The dictyonal strands are easily observed, they have diameter 0.09–0.12 mm, beams between the strands are 0.03–0.07 mm in diameter. Free rays of the dictyonal strands are protruded atrially. The meshes between the dictyonal strands and their connecting beams are rather regular, usually rectangular, 0.3–0.5×0.5–0.8 mm. Adjacent hexactinic spicules located among the dictyonal strands are irregularly and sparsely distributed among their meshes, they are connected to the framework by a single ray (small hexactins with rays 0.07–0.12/0.003–0.006 mm) and often at points of mutual contact (large hexactins with rays about 0.5/0.012–0.018 mm). The meshes there are very irregular and of different sizes. The dictyonal strands may be also observed in the vicinity of dermal surface but due to numerous epirhyzes, they are not straight as those from the atrial surface.

**Loose spicules:** dermal and atrial pentactins are similar to each other, they always have a rudiment about 0.02 mm long instead of the ray directed outside the body, rough surface, their outer ends are clavate, rounded, lanceolate or sometimes conically pointed. Tangential rays of dermal pentactins are 0.102–0.432 mm long (Table 1), the ray directed inside the body is 0.048–0.258 mm long (Table 1), the diameter of these rays is 0.002–0.009 mm. Tangential rays of atrial pentactins are 0.078–0.372 mm long, ray directed inside the body is 0.036–0.342 mm long (Table 1), the diameter of these rays is 0.004–0.009 mm.

Microscleres are stellate discohexasters only, with 8–14 secondary rays. The diameter of the discohexaster is 0.025–0.046 mm, their primary rosette is 0.006–0.018 mm in diameter (Table 1).

**Figure 3. F3:**
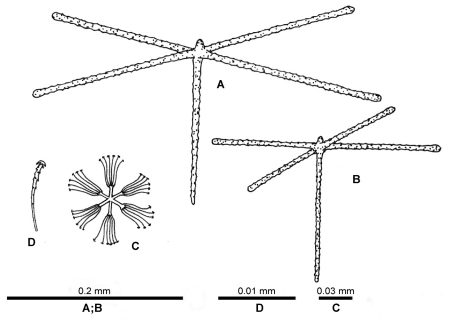
*Indeilla* gen n. *ridgenensis* sp.n. drawings of spicules of the holotypes **A** dermal pentactin **B** atrial pentactin, **C** discohexaster **D** secondary ray of discohexaster

**Figure 4.**
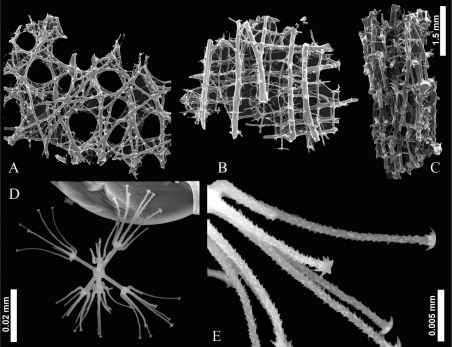
Scaning Electron Microscopy of *Indeilla* gen. n. *ridgenensis* sp. n. Frameowrk and spicules of the holotypes **A** dermal layer **B** atrial layer **C** lateral view **D** discohexaster **E** secondary ray tuft of discohexaster

**Table 1.** Spicule dimensions of *Indiella* gen. n. *ridgenensis* sp. n. (in mm). L - length, D - diameter, d - diameter of a primary rosette (N = number of observations; Min = minimum; Max = maximum; Avg = average; SD = standard diviations). Bold measurements are used in the text sections.

**Type**		**L Dermal pentactin**		**L Atrial pentactin**		**Discohexaster**	
		**Tangential ray**	**Ray directed inside body**	**Tangential ray**	**Ray directed inside body**	**D discohexaster**	**d discohexaster**
**Holotype**	N	25.000	25.000	25.000	25.000	28.000	28.000
	Min	**0.102**	0.060	**0.078**	0.042	**0.025**	**0.006**
	Max	**0.432**	**0.258**	0.360	**0.342**	**0.046**	**0.018**
	Avg	0.280	0.103	0.228	0.100	0.039	0.012
	SD	0.099	0.048	0.081	0.067	0.005	0.003
**Paratype-1**	N	16.000	16.000	12.000	12.000	14.000	14.000
	Min	0.168	**0.048**	0.108	0.060	0.032	0.009
	Max	0.408	0.180	0.360	0.156	0.042	0.018
	Avg	0.256	0.113	0.264	0.115	0.037	0.012
	SD	0.070	0.049	0.076	0.032	0.003	0.002
**Paratype-2**	N	7.000	7.000	6.000	6.000	3.000	3.000
	Min	0.240	0.078	0.132	0.048	0.039	0.012
	Max	0.414	0.192	**0.372**	0.114	0.041	0.014
	Avg	0.348	0.127	0.241	0.075	0.040	0.013
	SD	0.060	0.039	0.095	0.023	0.001	0.001
**Paratype-3**	N	5.000	5.000	4.000	4.000	1.000	1.000
	Min	0.168	0.072	0.150	**0.036**	0.032	0.008
	Max	0.312	0.168	0.240	0.084	0.032	0.008
	Avg	0.252	0.110	0.197	0.066	0.032	0.008
	SD	0.067	0.037	0.038	0.021	-	-

##### Remarks.

 Since all these fragments of the holotype and of paratypes were collected from the same station, there is a great probability that they belong to a single specimen.

### Key to the Genera of Aulocalycidae

**Table:** 

1	Dictyonal strands not obvious, likely entirely absent (if present they are distributed chaotically), choanosomal hexactins fuse at points of mutual contacts, their distal and proximal rays are bent in the tangential plane (aulocalycoid skeleton)	2
–	Dictyonal strands present in addition to aulocalycoid skeleton, dictyonal strands are distributed in common, longitudinal direction	5
2	Body of branching tubes or cup with short lateral tubes	3
–	Body fan- or tongue-shape without tubular elements	*Euryplegma*
3	With rhopalasters as distinctive microscleres	*Aulocalyx*
–	Without rhopalasters	4
4	Parietal gaps large and closely spaced; wall lace-like	*Rhabdodictyum*
–	Parietal gaps small, sparse; wall thin and mostly imperforate	*Ijimadictyum*
5	Walls unchannelized	*Leioplegma*
–	Walls channelized by epirhyses	*Indiella* gen. n.

**Remarks.** It is not obvious that the genus *Euryplegma* has schizorhyses, as postulated in the key of genera by Reiswig (2002); a possibility of lateral oscula and cavaedia (Tabachnick and Reiswig 2000) cannot be rejected. This newly suggested version of the key to genera of Aulocalycoidae family avoids this problem.

## Supplementary Material

XML Treatment for Indiella

XML Treatment for Indiella ridgenensis
